# Integration of Error Compensation of Coordinate Measuring Machines into Feature Measurement: Part I—Model Development

**DOI:** 10.3390/s16101610

**Published:** 2016-09-29

**Authors:** Roque Calvo, Roberto D’Amato, Emilio Gómez, Rosario Domingo

**Affiliations:** 1 Department of Mechanical Engineering, Chemistry and Industrial Design, Universidad Politécnica de Madrid, Ronda de Valencia 3, Madrid 28012, Spain; r.damato@upm.es (R.D.); emilio.gomez@upm.es (E.G.); 2 Department of Construction and Manufacturing Engineering, Universidad Nacional de Educación a Distancia (UNED), C/Juan del Rosal 12, Madrid 28040, Spain; rdomingo@ind.uned.es

**Keywords:** CMM uncertainty, CMM error mapping, CMM verification, flatness measurement, angle measurement, roundness measurement

## Abstract

The development of an error compensation model for coordinate measuring machines (CMMs) and its integration into feature measurement is presented. CMMs are widespread and dependable instruments in industry and laboratories for dimensional measurement. From the tip probe sensor to the machine display, there is a complex transformation of probed point coordinates through the geometrical feature model that makes the assessment of accuracy and uncertainty measurement results difficult. Therefore, error compensation is not standardized, conversely to other simpler instruments. Detailed coordinate error compensation models are generally based on CMM as a rigid-body and it requires a detailed mapping of the CMM’s behavior. In this paper a new model type of error compensation is proposed. It evaluates the error from the vectorial composition of length error by axis and its integration into the geometrical measurement model. The non-explained variability by the model is incorporated into the uncertainty budget. Model parameters are analyzed and linked to the geometrical errors and uncertainty of CMM response. Next, the outstanding measurement models of flatness, angle, and roundness are developed. The proposed models are useful for measurement improvement with easy integration into CMM signal processing, in particular in industrial environments where built-in solutions are sought. A battery of implementation tests are presented in Part II, where the experimental endorsement of the model is included.

## 1. Introduction

CMM flexibility to cope with the measurement of complex geometries is based on point coordinate determination and the geometry reconstruction through computer algorithms. The basic measurement process is the coordinate of the physical point on a coordinate reference system by contact (touch probe sensor) or contactless (optical sensor) means. The CMM’s raw measurand is, directly, the point coordinates. In an ordinary direct measurement, the methodology includes sampling the measurand, estimating the best expected value (statistical mean of the sample) and the associated uncertainty to this estimation. The ISO guide to the expression of uncertainty (GUM) is a globally-adopted standard. It includes two main methodologies. For most of the direct measurements, a first basic frequentist approach is to consider the uncertainty as the standard deviation of the mean through the standard deviation of the sample. Nevertheless, many measurements are not direct. Indirect measurement of simple dimensional measurands or tolerance of the form needs a conversion of the input values through a measurement model. For complex measurement models, and based on the coordinate uncertainty as input, the Monte Carlo method allows uncertainty estimation through intensive calculation. Thus, an ulterior verification of the assumed uncertainty model of the coordinates against a known standard is necessary to provide likelihood to the model [1].

In the case of a CMM as a measuring instrument, the complexity of the measurement chain increases by the dynamical process of signal acquisition and the movement of the measuring head. Next, the indicated coordinates are processed through algorithms of geometry substitution to propose a measurement result.

**Figure 1:**
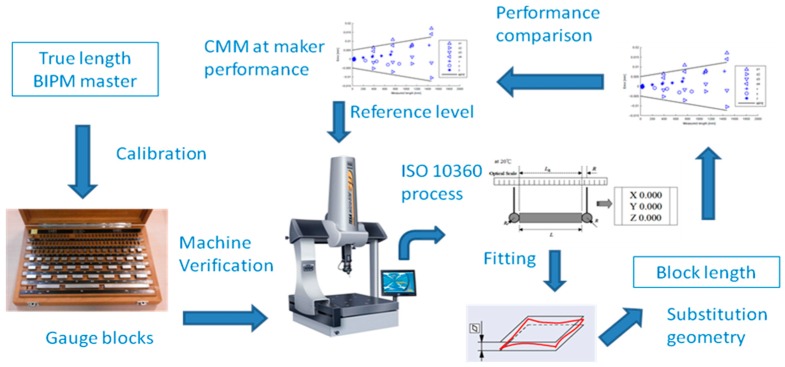
Sketch of a CMM verification process by ISO 10360-2.

CMMs do not meet the Abbé principle of measuring practice because the direction of measurement is not the extension of the scale that offers the coordinate [2]. Ordinary CMMs have three mutually orthogonal scales. In addition to the static small deviations from the machine construction, the dynamics of the carriages’ displacement creates deviation of the probe from the theoretical positions. Therefore, it seems to be a major task to account for a full traceability of any deviation through the complete and complex chain of measurement of a CMM. Even when the formal expression of measurement requires its best value and its uncertainty, enforcing a rigorous treatment of measurement output can be useless and of little productivity in industrial environments. Therefore, an overall CMM error bounding is a standard approach to complement the best value of measurement (see Figure 1) by ISO 10360-2 [3]. CMM’s acceptance or verification seems to be at the borders of the ordinary metrology chain of calibration by introducing overall error estimations. Nonetheless, international standards require the estimation of uncertainty in the expression of any measurement, in particular to comply with ISO/IEC 17025 [4]. In addition, the development of ISO 15530-3 [5] deals with CMM uncertainty estimation.

This work is presented in two parts. In Part I a model of error is developed following some standard CMM verification techniques. In Section 2 basic considerations of CMM measurement are introduced to approach the proposed CMM error model. In Section 3 the proposed basic error model of length is developed. Section 4, Section 5 and Section 6 include the models for flatness, angle, and dihedral angle, and roundness measurement with the interpretation analysis of the CMM model in Section 7. In Part II the experimental implementation is carried out and discussed. In its Section 2 the experimental error mapping from a standard verification test of a moving bridge CMM is included. Then, in Section 3, the experimental results and remarks of the model interpretation are presented. In Section 4 the experimental trials with calibrated artefacts are analysed. Parts I and II both include overall concluding remarks.

## 2. Approaches to CMM Errors

The ISO 10360 series offer a consolidated framework to CMM manufacturers and the machine owners to accept and verify CMMs. They do not provide further insights to improve measurement results. In practical terms, it allows the expression of machine indication plus or minus the overall estimated error bound, which becomes an interval of uncertainty. The efforts to obtain a more precise expression of CMM’s measurements should deal with a more detailed error description to correct CMM indication. Error compensation (in the volume of work) is based on sampling calibrated artefacts. The repeatability of the results from a series of measurements of the same physical position (point) is a direct demonstration of the uncertainty associated with the measurement capability of the CMM.

In addition to a good measurement practice in a stable environment (particularly temperature control), the attempts to model the expected deviations of a CMM under repeatability conditions in the literature are mostly through vectorial composition. They include the mechanics of deviations from combined translations and rotations of the point coordinates and a kinematic rigid body model [6,7,8]. Once those deviations are known, a more precise position of the point coordinates can be obtained, so a more precise determination of the measurand is expected.

The violation of the Abbé principle by CMMs limits the capability of improving the precision of CMMs via hardware [9]. Alternatively, error compensation via signal processing or measurement post-processing can be a convenient improvement method to reach the precise point coordinate in both CMMs and computer numerical control (CNC) devices [10,11]. It is important to note that the former initiatives, and many others pursuing error compensation of point coordinates, are based on the correction of the coordinate position and a kinematic error model [12,13,14,15]. The present work has the advantages of considering the distance between points to correct average errors. Some previous initiatives that consider the differences of coordinates between points for error evaluation involve the classic reversal techniques [16], in the early attempts to deal with error [16], and obviously in the standard verification tests by the ISO 10360-2 series, or the similar ASME B89.4.10360.2 [3,4,5,6,7,8,9,10,11,12,13,14,15,16,17,18]. In these former cases the measurand verification is the length of calibrated gage blocks, not the point coordinates themselves.

As is known, the Euclidean distance L2 is the metric of the distance between two points. Any measurement on a CMM is initially based on the relative position in space between the point coordinates. This relative position can be equated through the distance between the points. The absolute position of points (the conventional assigned coordinate value in the space with respect to the CMM, in metrological terms) has a relative importance as long as it provides the correct distance between the points on the specimen under measurement. This relative position importance is also present in the measurement origin in the CMM scales that could be arbitrarily zeroed. Either way, the relative position of the origin is present in the scales in computer numerical control (CNC) systems without the theoretical impact on final measurement or machining results. The ordinary CMM model of coordinate points corresponds to the Euclidean space. The proposed error model lays mainly on the difference of coordinates, which define a vector with modulus and direction. Regardless of the origin of the reference system, this structure corresponds with the concept of affine space.

## 3. CMM Error Model of Length

CMM performance verification by ISO 10360-2 and -5 [19] includes the estimation of three basic magnitudes: a maximum permissible error of length *E_L,MPE_*, a maximum permissible error of the probe E_P,MPE_, and a repeatability estimation R. Those are probabilistic intervals evaluated ordinarily at a 95% confidence level, while R is evaluated from its maximum. In length measurement, the ISO model applies the overall *E_L,MPE_* over the value of the machine provided that all known biases (systematic errors) are previously corrected, *L_unbiased_*. In the CMM verification process the deviation of every length indicated by the machine is checked to ensure that they do not surpass ±*E_L,MPE_*, with respect to the assigned values of the gage blocks. Next, in the ordinary CMM process of measurement, *E_L,MPE_* can be incorporated into the uncertainty budget (other effects could be aggregated by the law of uncertainty propagation). The best value of the length of measurement *L_m_* can be expressed from the indication and an uncertainty estimation by Equation (1):

(1) $$L_{m}=L_{unbiased}\pm E_{L,MPE}(L)$$

**Figure 2:**
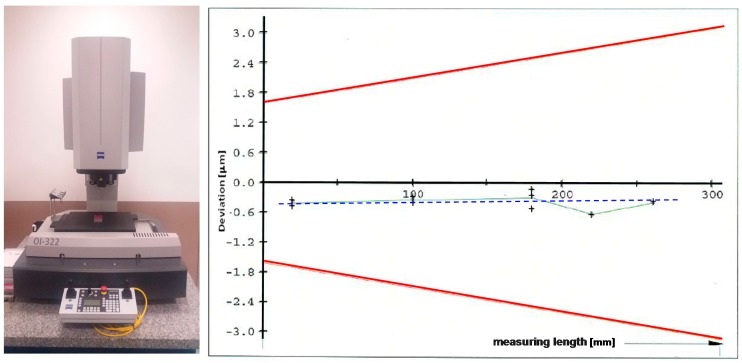
First-order linear trend by axis of a verification test by ISO 10360 (X-axis). CMM model O-Inspect 322 by Carl Zeiss Industrielle Messtechnik GmbH, Oberkochen, Germany, measurement field XxYxZ of 300 mm × 200 mm × 200 mm, including probe head VAST XXT(LT1), and measurement software CALYPSO 2014. Red lines are the control limits at 95% confidence level that establish the maximum permissible error.

A new model of error aggregation is proposed. It considers a linear relationship of the error by each of the three main axes with the indication of the CMM, while independently measuring by every axis direction. The gage blocks also have an uncertainty (type B) that typically grows linearly with their nominal length and the grade. In addition, related with the CMM construction itself, the error associated with the CMM scales has an amplification effect on the error associated directly with the total length under measurement [1,13,20,21]; see, also, a case example in Figure 2. Therefore, this linear relationship is a priori alike and it will be verified experimentally for a common CMM machine in Part II of this work. Denoted by *L_mx_*, the best value for the measurand length aligned with the X axis is meant, for *L_x_*, the CMM indication, and for *L_nx_*, the former indicated length after mean error correction. The mean error E in the formulae is defined as the assigned value of the length standard (calibrated gage) minus the measured value, unlike the standard International Vocabulary of Metrology (VIM) definition (difference between the measured quantity value minus the conventional value of reference) [22]. This notation is adopted just for convenience of signs and clearer formulae, but with no effect in the overall results. Finally, ±*Û_x_*(*L_x_*) indicates an interval of prediction (at a level of confidence) of the maximum error when measuring under repeatability conditions by the X axis, where ±*Û_x_* is an upper overall estimation for the full field of measurement. The same notation for Y and Z axes, mutatis mutandis, allows expressing the best value for the length of the measurand aligned by each axis, after Equation (2):

(2) $$\begin{array}{l} L_{mx}=L_{x}+E_{x}\pm {\hat{U}}_{x}\left(L_{x}\right)=L_{x}+(A_{x}+B_{x}\cdot L_{x})\pm {\hat{U}}_{x}=L_{nx}\pm {\hat{U}}_{x}\\ L_{my}=L_{y}+E_{y}\pm {\hat{U}}_{y}\left(L_{y}\right)=L_{y}+(A_{y}+B_{y}\cdot L_{y})\pm {\hat{U}}_{y}=L_{ny}\pm {\hat{U}}_{y}\\ L_{mz}=L_{z}+E_{z}\pm {\hat{U}}_{z}\left(L_{z}\right)=L_{z}+(A_{z}+B_{z}\cdot L_{z})\pm {\hat{U}}_{z}=L_{nz}\pm {\hat{U}}_{z} \end{array}$$

The *Û* resembles uncertainty notation, as in fact it is an estimation from the measurement sampling, in addition to other uncertainty contributions of temperature, operator, etc. The prediction bounds at 95% confidence level of the spread around the mean value of the model will be identified as the non-explained variability of the regression model of error by axis. The overall *E_L,MPE_* can be considered an estimation of the expanded uncertainty (k = 2) of the CMM [6]. The expected original verification of the maximum permissible error specified by ISO 10360-2 [3] aims at testing that errors are contained between the limits of the performance specification. Nevertheless, the verification test techniques of ISO 10360-2 with calibrated artefacts under repeatability conditions could be used for assigning values to the errors in those conditions. For the proposed model those conditions are not any rated operating conditions allowed by ISO 10360-2, but the reference operating conditions [23] that will allow the use of the model results under similar measurement conditions for feature measurement correction. In this task of assigning values to the errors of the CMM to improve measuring results, all other sources of measurement uncertainty to the CMM uncertainty in the test conditions *Û* [6] should be added. In the case of a verification test, the machine compliance in any direction is sought. The direction of measurement is part of the uncontrolled factor, and the uncertainty of the CMM is an overall estimation regardless the direction of measurement and in the range of operative conditions of the machine. Note that fixing the *E_L,MPE_* at 95% confidence for the standard verification test ISO 10360-2 departs from a regression of errors at each length in any direction, even when, in general, a measurement is taken in a vectorial way, just in a predefined direction by the normal to the physical surface. The overall error by the ISO 10360-2 model does not discriminate the direction of measurement. As a result, the errors sampled through this verification model are useful for conformance of machine performance limits, but *E_L,MPE_* can overestimate CMM behaviour in particular directions.

In the proposed model, the contribution of measuring in the direction of measurement is considered by axis, separated and independent from each other, but under the same reference operating conditions. The direction of the measurement normal to the surface of the specimen appears in the feature measurement model and incorporates the scalar components by the axis of the model. The experimental results of Part II will support a stronger functional relationship of errors by axis. Nevertheless, no covariance or mutual influence between axis errors is considered by the proposed model. This will be discussed in Section 5 to support the proposed approach.

The Euclidean distance between two points is, in fact, the brick of a measurement model construction that puts in relationship the point coordinates with the measurement length *L_m_* of a dimension, in an arbitrary direction of the volume of measurement CMM, by Equation (3), where bold symbols denote vectors:

(3) $$\begin{array}{cc} {L^{2}}_{m} & ={L^{2}}_{mx}+{L^{2}}_{my}+{L^{2}}_{mz}={\left[L_{nx}\pm {\hat{U}}_{x}\right]}^{2}+{\left[L_{ny}\pm {\hat{U}}_{y}\right]}^{2}+{\left[L_{nz}\pm {\hat{U}}_{z}\right]}^{2}\\ & ={L_{nx}}^{2}+{L_{ny}}^{2}+{L_{nz}}^{2}\pm 2L_{nx}{\hat{U}}_{x}\pm 2L_{ny}{\hat{U}}_{y}\pm 2L_{nz}{\hat{U}}_{z}+O(\hat{U})\\ & \approx {L_{n}}^{2}\pm 2L_{nx}{\hat{U}}_{x}\pm 2L_{ny}{\hat{U}}_{y}\pm 2L_{nz}{\hat{U}}_{z}={L_{n}}^{2}\pm 2\hat{U}\cdot L_{n}\\ & \text{as providing ordinary measuring capability}\;{Û}_{x},{Û}_{y},{Û}_{z},Û<<L_{x},L_{y},L_{z},L\\ & \begin{array}{cccc} \text{where}, & \hat{U}=\left({\hat{U}}_{x},{\hat{U}}_{y},{\hat{U}}_{z}\right); & L_{n}=\left(L_{nx},L_{ny},L_{nz}\right); & \hat{U}=\sqrt{{{\hat{U}}_{x}}^{2}+{{\hat{U}}_{y}}^{2}+{{\hat{U}}_{z}}^{2}}; \end{array}\\ {L_{n}}^{2} & =L_{n}\cdot L_{n}={L^{2}}_{nx}+{L^{2}}_{ny}+{L^{2}}_{nz}={\left(A_{x}+(B_{x}+1)\cdot L_{x}\right)}^{2}+{\left(A_{y}+(B_{y}+1)\cdot L_{y}\right)}^{2}+{\left(A_{z}+(B_{z}+1)\cdot L_{z}\right)}^{2} \end{array}$$

This allows identifying the uncertainty estimated by the maximum permissible error of Equation (1) in a first-order approach by Equation (4). Therefore, *E_L,MPE_* could be expressed through the proposed model as the projection of a vectorial uncertainty U on the measurement direction. Of note, this direction is determined by the corrected coordinates of the points, (*A_x_* + (*B_x_* + 1))*L_x_*, see Equation (6), which is applicable instead of the length from CMM indication *L_x_*.

(4) $$\begin{array}{l} {L^{2}}_{m}={\left[L_{n}\pm \hat{U}\right]}^{2}\approx {L_{n}}^{2}\pm 2L_{n}\cdot \hat{U}\\ \text{as providing}\;{Û}_{x},{Û}_{y},{Û}_{z},Û<<L_{x},L_{y},L_{z},L,\;\text{the Taylor series development allows expressing}\\ L_{m}\approx \sqrt{{L^{2}}_{n}\pm 2\hat{U}\cdot L_{n}\cdot }=L_{n}\sqrt{1\pm \frac{2\hat{U}\cdot L_{n}}{{L^{2}}_{n}}}\approx L_{n}\left[1\pm \frac{1}{2}\left(\frac{2\hat{U}\cdot L_{n}}{{L^{2}}_{n}}\right)\right]=L_{n}\pm \frac{\hat{U}\cdot L_{n}}{L_{n}};\;if\;\hat{U}\cdot L_{n}<<{L^{2}}_{n} \end{array}$$

The error correction and uncertainty, from CMM indication, in an arbitrary direction can be expressed by Equation (5):

(5) $$\begin{array}{l} L_{m}=L+E\pm {\hat{U}}_{m}=L_{n}\pm {\hat{U}}_{m};\\ {\hat{U}}_{m}=\frac{\hat{U}\cdot L_{n}}{L_{n}};\;E=L_{n}-L;\;\hat{U}=\left|\hat{U}\right|=\sqrt{{{\hat{U}}_{x}}^{2}+{{\hat{U}}_{y}}^{2}+{{\hat{U}}_{z}}^{2}} \end{array}$$

Finally, term identification between Equation (4) and the first statement of Equation (5) gives the mean error correction *E* and *Û_m_* expression function of the contributions by each axis of the CMM, Equation (6). Of note, the uncertainty estimation from sampling the measurement *L_m_* is *Û_m_* and it appears as the vectorial projection of the vector of uncertainty (non-explained variability) by each axis *Û* in the direction of distance measurement (by the normal direction to the surface) given by the vector of the corrected length *L_n_*. The expression of the uncertainty scalar *Û* resembles the law of uncertainty propagation for the independent contributions by each axis, following GUM. In fact, both the proposed model in Equation (3) and the derivation of the uncertainty (in a first-order approach) use the Taylor series to derive the expression. The proposed measurement model comes directly from the Euclidean distance from the point coordinates and a Taylor series expansion of the expression. The GUM law of uncertainty propagation uses the Euclidean distance to define the spread of a measurement by the Taylor expansion of its variance. This is given by the sum of squares of the distance of the measurement result to the average result [24]. A comparison of the uncertainty by the law of uncertainty propagation and this model will be carried out in Section 7.

(6) $$\begin{array}{l} \begin{array}{cc} {\hat{U}}_{m}= & \frac{\hat{U}\cdot L_{n}}{L_{n}}=\left|\frac{{\hat{U}}_{x}(A_{x}+(B_{x}+1)\cdot L_{x})+{\hat{U}}_{y}(A_{y}+(B_{y}+1)\cdot L_{y})+{\hat{U}}_{z}(A_{z}+(B_{z}+1)\cdot L_{z})}{\sqrt{{\left(A_{x}+(B_{x}+1)\cdot L_{x}\right)}^{2}+{\left(A_{y}+(B_{y}+1)\cdot L_{y}\right)}^{2}+{\left(A_{z}+(B_{z}+1)\cdot L_{z}\right)}^{2}}}\right|=\left\|\hat{U}\cdot n\right\|\\ & \hat{U}=\left({\hat{U}}_{x},{\hat{U}}_{y},{\hat{U}}_{z}\right);\;U=\sqrt{{{\hat{U}}^{2}}_{x}+{{\hat{U}}^{2}}_{y}+{{\hat{U}}^{2}}_{z}}\\ & n=L_{n}/\left\|L_{n}\right\|;\;L_{n}=\left(A_{x}+(B_{x}+1)\cdot L_{x},\;A_{y}+(B_{y}+1)\cdot L_{y},\;A_{z}+(B_{z}+1)\cdot L_{z}\right) \end{array}\\ \begin{array}{ccc} E=L_{n}-L=L_{n}-\sqrt{{L^{2}}_{x}+{L^{2}}_{y}+{L^{2}}_{z}}; & & L_{m}=L_{n}\pm {\hat{U}}_{m}=\sqrt{{L^{2}}_{x}+{L^{2}}_{y}+{L^{2}}_{z}}+E\pm {\hat{U}}_{m} \end{array} \end{array}$$

The proposed length model (Equations (5) and (6)) expresses the measurement result as the nominal calculation from the CMM indication, plus a correction with confidence bounds in an interval determined by the projection of the vector of estimated uncertainty by axis *Û* by the direction of the measurement after correction, defined by *L_n_*.

Formally, the use of this disaggregated model allows considering the limit case of *L* → 0. The interpretation of *Û_m_* corresponds to an estimation of the point uncertainty, whose value is the uncertainty by the law of uncertainty propagation for independent contributions, but projected by the direction of the measurement after correction, by Equation (7). In this limit case, this direction is determined by the direction of the vector *E_0,MPE_,* the vectorial composition of the contributions by each axis direction:

(7) $$\begin{array}{ccc} L\to 0 & \Rightarrow & {\hat{U}}_{m}\approx \frac{{\hat{U}}_{x}A_{x}+{\hat{U}}_{y}A_{y}+{\hat{U}}_{z}A_{z}}{\sqrt{{A^{2}}_{x}+{A^{2}}_{y}+{A_{z}}^{2}}}=\hat{U}\cdot n=\sqrt{{{\hat{U}}_{x}}^{2}+{{\hat{U}}_{y}}^{2}+{{\hat{U}}_{z}}^{2}}\cdot \text{cos}(\hat{U}\cdot n)\\ & & \hat{U}=\left({\hat{U}}_{x},{\hat{U}}_{y},{\hat{U}}_{z}\right);\;n=L_{n}/\left\|L_{n}\right\|=\frac{\left(A_{x},A_{y},A_{z}\right)}{\sqrt{{A^{2}}_{x}+{A^{2}}_{y}+{A_{z}}^{2}}};\\ & & \text{where}\;E_{0,\text{MPE}}=\left(A_{x},A_{y},A_{z}\right)\;\text{and}\;E_{0,MPE}=\left|E_{0,\text{MPE}}\right|=\sqrt{{A^{2}}_{x}+{A^{2}}_{y}+{A_{z}}^{2}}\\ L\to 0 & \Rightarrow & E=L_{n}-L;\;E\to \sqrt{{A^{2}}_{x}+{A^{2}}_{y}+{A_{z}}^{2}} \end{array}$$

In the proposed model this residual uncertainty of the point is the irreducible reproducibility of the point coordinates regardless of the length under measurement. This could allow a possible interpretation as an estimation of the error of the true position of the point. The coordinate errors in CMM lay in the error of indication with respect to the true value with the origin in up to 21 types of error components affecting the coordinate determination [14]. Successive measurement repetitions on the same physical point in a unilateral measuring *E_0,MPE_* shows the spread of CMM repeatability. The mean value could be biased with respect to the true value, and it could be the origin of a net bias (systematic error) in the determination of the length of a calibrated standard by the CMM, even when using a significant sample size under repeatability conditions. In the case of a complete correction of the CMM coordinate indication by the manufacturer, of any bias in any point and in any direction, this bias would not exist, and the repeatability would become simply the uncertainty of the CMM indication of null correction. Nonetheless, the proposed model takes into consideration the direction of measurement; therefore, a better allocation of the original source of errors at every point is expected with the purpose of error assignation, which certainly can depend on the direction of measurement.

An important theoretical question arises from the concept of error as a quantity. The CMM model in ISO 10360-2 considers the error as a quantity value from the difference of two values: the CMM indication and a reference value (the conventional assigned value of the length gage block length). Therefore, once all known bias (systematic errors) in the indication are compensated by the manufacturer, the maximum permissible error is a quantity value that bounds an interval of confidence or uncertainty. The errors found in the verification test are samples in the range of operative conditions of the machine. That is, in fact, the nature of uncertainty in ordinary direct, unbiased measurements, so after all systematic error correction the error appreciated in the sample is random around a null mean. The bias or systematic error is a quantity value under GUM, and it does not have a probability density function to support it as a quantity [25]. The difference of quantity and quantity value is also analysed in [26], so in the CMM verification approach by ISO 10360-2, the overall estimation of error by the maximum permissible error is also considered a quantity value. The differences with respect to the standard are considered for multiple directions of measurement or the rated operating conditions, so that there is no spatiotemporal address [25] in such an average of values bounded by *E_L,MPE_*. Only three repetitions of every measurement are taken, without the objective of evaluating a mean error. It is just a quantity value adopted to define a confidence interval, and not a magnitude subject to possible measurement itself. ISO 23165 [27] develops test uncertainty for a CMM as an indicating instrument, complementary of the error bounds estimated by ISO 10360-2, in accordance with ISO 14253-1 [28]. It is focused on the uncertainty in the verification test by ISO 10360-2. Both ISO standards establish the framework for CMM acceptance and performance verification between the specified limits.

In the proposed model, CMM error is disaggregated into the quantity error of mean error by axis and a quantity value from the spread around the mean error that will contribute to the uncertainty of the feature measurement by the CMM. It follows the evolving considerations of error in [25,26]; therefore, in the proposed model the error by axis is treated as a quantity based on experimental evidence of machine behaviour by axis in concrete conditions of testing. As a consequence, it is possible to consider the uncertainty of error by axis and its contribution to the uncertainty budget under the reference operating conditions. The error is treated as a quantity: in fact, it is determined by vectorial composition from each contribution of error by axis based on the regression of a distribution of values measured by axis. Next, the error and its uncertainty will be incorporated into the model of the feature measurement simultaneously through vectorial calculus. The linear regression model is the simplest and more desired model to deal with the machine behaviour, but this conceptual approach could be also valid for other machine responses. In order to be a useful model, the measurement reference operating conditions of the tests for error assignation should be similar enough to those used later in the measurement of the feature in the specimen. This requirement approximates those imposed in the non-substitution estimation of uncertainty by ISO 15530-3 [5] from calibrated artefacts. The uncertainty of the proposed model of length can be identified with the standard uncertainty due to the measurement process in a non-substitution procedure in the operating conditions of reference. Furthermore, the basic proposed model of error and uncertainty for length can be incorporated into the measurement of other features, so it allows integrating the results from calibrated blocks into more complex measurands.

## 4. CMM Error Model of Flatness

**Figure 3:**
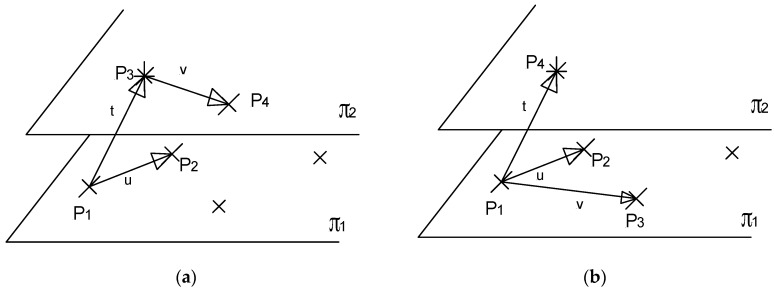
Flatness under minimum zone tolerance criteria. (**a**) configuration (2–2); (**b**) configuration (3–1).

The form tolerance of flatness is determined by four non-coplanar points for any finite set of points, regardless of its size [29]. By each pair, two non-colinear directions are determined, and the flatness is measured by their common normal direction and minimum distance between both planes, Figure 3. Obviously, when three of them are coplanar, the distance of the fourth to the plane becomes the flatness measure. The algorithm for accurate calculation of flatness from a set of point coordinates can be found in [29].

Starting from the CMM indication, the vectorial calculation of the minimum zone flatness (MZF) from the three vectors u(p1,p2), v(p3,p4), and t(p1,p3) is given by Equation (8), where the mix product can be developed from the determinant of the matrix built by the difference of coordinates.

(8) $$\begin{array}{l} MZF_{uvt,indication}=\left|\frac{\left(u\times v\right)\cdot t}{\left\|u\times v\right\|}\right|=\left|\frac{1}{\left\|u\times v\right\|}\cdot \text{det}\left[\begin{array}{ccc} x_{3}-x_{1} & y_{3}-y_{1} & z_{3}-z_{1}\\ x_{2}-x_{1} & y_{2}-y_{1} & z_{2}-z_{1}\\ x_{4}-x_{3} & y_{4}-y_{3} & z_{4}-z_{3} \end{array}\right]\right|\\ u=(x_{2}-x_{1},y_{2}-y_{1},z_{2}-z_{1});\;v=(x_{4}-x_{3},y_{4}-y_{3},z_{4}-z_{3});\;t=(x_{3}-x_{1},y_{3}-y_{1},z_{3}-z_{1}) \end{array}$$

In Equation (8) the coordinates are directly the indication of the CMM. The error of a length is the error of the absolute value of the difference of coordinates or the distance between points, for the x axis, *E_x_* = *E_x_*(*L_x_*) and *L_x_* = |x*_i_* − *x_j_*|. Considering a proper resolution and measurement capability, errors and uncertainty are expected to be much smaller than the distance between points. The expression of the measured minimum zone flatness (*MZF_m_*) to the leading order can be developed with Equation (9).

This error model can be applied for the flatness results of both the least-squares and the minimum zone tolerance criteria, but the critical points in the envelope planes will be four in the case of the minimum zone and, in general, two different ones in the case of least-squares. Therefore, the quantitative results will be also different depending on the flatness criteria. It is significant in the model that the error of flatness is a direct consequence of the absolute value of coordinate difference (distance between points) errors, where the primitive source of error and uncertainty can be found. Equation (9) is applied just for a measurement. Like the measurement of length, a reliable measurement of flatness should be based in the most probable results obtained from several samples. Considering that the expression of the minimum zone tolerance can be obtained from four points, at least one on each envelope plane, four different combinations of points are possible to define u,v,t from them (see Figure 2) in the configuration (2–2), and up to six possible estimations can be derived for error and uncertainty estimation in the configuration (3–1) from the four critical points. The best value of flatness from the indication, *MZF_uvt_* in Equation (9), will be the same in a first-order approach but, in general, the error and the uncertainty of every configuration will be different.

(9) $$\begin{array}{l} MZF_{m,uvt}=\left|\frac{u_{m}\times v_{m}}{\left\|u_{m}\times v_{m}\right\|}\cdot t_{m}\right|\approx \left|\frac{\left(u\times v\right)\cdot \left(t+E_{t}\right)+\left[\left(u\times E_{v}\right)\cdot \left(t+E_{t}\right)+\left(E_{u}\times v\right)\cdot \left(t+E_{t}\right)\right]}{\left\|\left(u+E_{u}\right)\times \left(v+E_{v}\right)\right\|}\right|\\ \pm \left|\frac{\left[\left(\left(u+E_{u}\right)\times {\hat{U}}_{v}\right)\cdot \left(t+E_{t}\right)+\left({\hat{U}}_{u}\times \left(v+E_{v}\right)\right)\cdot \left(t+E_{t}\right)+\left(\left(u+E_{u}\right)\times \left(v+E_{v}\right)\right)\cdot {\hat{U}}_{t}\right]}{\left\|\left(u+E_{u}\right)\times \left(v+E_{v}\right)\right\|}\right|=MZF_{uvt}+E_{uvt}\pm {\hat{U}}_{uvt}\\ \;\;\;\;\;\;\;\;\;u_{m}=u+E_{u}\pm {\hat{U}}_{u}=(x_{2}-x_{1}+E_{x12}\pm {\hat{U}}_{x12},y_{2}-y_{1}+E_{y12}\pm {\hat{U}}_{y12},z_{2}-z_{1}+E_{z12}\pm {\hat{U}}_{z12});\\ \;\;\;\;\;\;\;\;\;v_{m}=v+E_{v}\pm {\hat{U}}_{v}=(x_{4}-x_{3}+E_{x34}\pm {\hat{U}}_{x34},y_{4}-y_{3}+E_{y34}\pm {\hat{U}}_{y34},z_{4}-z_{3}+E_{z34}\pm {\hat{U}}_{z34});\\ \;\;\;\;\;\;\;\;\;t_{m}=t+E_{t}\pm {\hat{U}}_{t}=(x_{3}-x_{1}+E_{x13}\pm {\hat{U}}_{x13},y_{3}-y_{1}+E_{y13}\pm {\hat{U}}_{y13},z_{3}-z_{1}+E_{z13}\pm {\hat{U}}_{z13});\\ MZF_{uvt}=\left|\frac{\left(u\times v\right)\cdot t}{\left\|u+E_{u}\right\|\cdot \left\|v+E_{v}\right\|}\right|\approx \left|\frac{\left(u\times v\right)\cdot t}{\left\|u\times v\right\|}\right|;\\ E_{uvt}=\frac{\left(u\times E_{v}\right)\cdot \left(t+E_{t}\right)+\left(E_{u}\times v\right)\cdot \left(t+E_{t}\right)+\left(u\times v\right)\cdot E_{t}}{\left\|\left(u+E_{u}\right)\times \left(v+E_{v}\right)\right\|};\\ {\hat{U}}_{uvt}=\left|\frac{\left(\left(u+E_{u}\right)\times {\hat{U}}_{v}\right)\cdot \left(t+E_{t}\right)+\left({\hat{U}}_{u}\times \left(v+E_{v}\right)\right)\cdot \left(t+E_{t}\right)+\left(\left(u+E_{u}\right)\times \left(v+E_{v}\right)\right)\cdot {\hat{U}}_{t}}{\left\|\left(u+E_{u}\right)\times \left(v+E_{v}\right)\right\|}\right| \end{array}$$

Of note, the calculation of uncertainty by GUM from the measurement function directly in the least-squares method also presents the issue of evaluating the sensitivity coefficients at the solution. Since, in general, no point belongs to the least-squares solution, an approximation using near-by points is used instead [29].

The proposed model considers error as a quantity, which is why the mean value of the error can be proposed as its best estimation. In addition, uncertainty estimation could be estimated by the mean uncertainty, giving the treatment of a random variable. A conservative alternative for uncertainty estimation as a quantity value is just to give the maximum value reached through the different configurations. Nevertheless, having at least four configurations, the uncertainty from the variability of the error as a quantity in Equation (10) is proposed accordingly, already discussed in the previous section, for small samples at least of a size of four, and based on the t-distribution with n−1 degrees of freedom. In the experimental Part II of this work, a comparison of the quantification of those options will be presented.

(10) $$\begin{array}{cc} MZF_{m}= & MZF+\frac{1}{c}\sum_{ii}^{c}E_{i}\pm 2\sqrt{\frac{c-1}{c-3}}\frac{s}{\sqrt{c}}=MZF+E\pm {\hat{U}}_{k=2};\\ & \;where\;s=\sqrt{\frac{{\left(E_{i}-\bar{E}\right)}^{2}}{c-1}};\;E=\frac{1}{c}\sum_{ii}^{c}E_{i} \end{array}$$

The fact that under minimum zone criteria more than one solution to the evaluation of error is generated can be considered an advantage instead of a drawback, because it allows the statistical treatment of error as a quantity from just one sample (set of points) in one measurement.

## 5. CMM Error Model of Angle Measurement

Angle measurement is based geometrically on the orientation of the features (lines or surfaces) that define the angle. The definition of angle in the affine space of points with a metric (Euclidean space) is the cosine of two vectors that define the directions of reference. Therefore, the radiant is considered a supplementary unit and the angle magnitude is indirectly measured from metric relationships.

**Figure 4:**
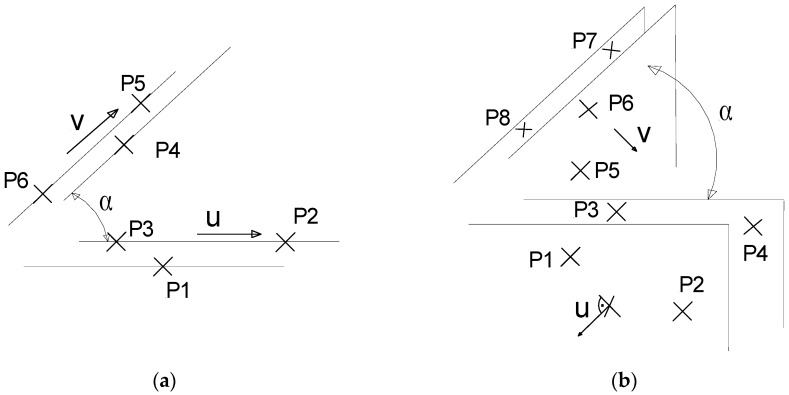
Minimum zone tolerance criteria of angle, (**a**) in the plane and (**b**) dihedral.

Length standards are realized in gage blocks, where their opposite surfaces are reference surfaces of measurement. In the same manner, angle standards are blocks that contain surfaces; therefore, the realization of the angle will involve the determination of the orientation of the block surfaces as a first step. Nevertheless, in the case of the projection of two reference planes onto a plane, their traces become straight lines on it. Therefore, the reference features for angle measurement are two straight lines, Figure 4. Additionally, in the case of contactless measurement on images, angles on a plane are features of interest. Straight lines are ideally defined by two points. Real straight contours differ from ideal ones in the form tolerance of straightness [29].

(11) $$\begin{array}{l} \alpha_{m}=\alpha +E_{\alpha }\pm {\hat{U}}_{\alpha }=\alpha_{n}\pm \hat{U}\\ \begin{array}{cc} \text{cos}\;\alpha_{m} & =\frac{u_{m}\cdot v_{m}}{\left\|u_{m}\right\|\cdot \left\|v_{m}\right\|}\approx \frac{u\cdot v}{\left\|u_{n}\right\|\cdot \left\|v_{n}\right\|}+\frac{u\cdot E_{v}+E_{u}\cdot v}{\left\|u_{n}\right\|\cdot \left\|v_{n}\right\|}\pm \frac{u\cdot {\hat{U}}_{v}+{\hat{U}}_{u}\cdot v}{\left\|u_{n}\right\|\cdot \left\|v_{n}\right\|}\\ & u_{m}=u+E_{u}\pm {\hat{U}}_{u}=(x_{2}-x_{1}+E_{x12}\pm {\hat{U}}_{x12},y_{2}-y_{1}+E_{y12}\pm {\hat{U}}_{y12});\\ & v_{m}=v+E_{v}\pm {\hat{U}}_{v}=(x_{4}-x_{3}+E_{x34}\pm {\hat{U}}_{x34},y_{4}-y_{3}+E_{y34}\pm {\hat{U}}_{y34}); \end{array}\\ \text{as providing}\;U<<1,\;\text{and in a first order approach to the leading order}\\ \begin{array}{cc} \text{cos}\alpha_{m}= & \text{cos}(\alpha_{n}\pm U_{\alpha })=\text{cos}(\alpha_{n})\text{cos}(U_{\alpha })\mp \text{sin}(\alpha_{n})\text{sin}(U_{\alpha })\\ & =\left[\text{cos}(\alpha_{n})\mp \text{sin}(\alpha_{n})\tan(U_{\alpha })\right]\text{cos}(U_{\alpha })\approx \text{cos}(\alpha_{n})\mp \text{sin}(\alpha_{n})U_{\alpha };\;so \end{array}\\ \alpha_{n}={\text{cos}}^{-1}\left[\frac{u\cdot v+u\cdot E_{v}+E_{u}\cdot v}{\left\|u+E_{u}\right\|\cdot \left\|v+E_{v}\right\|}\right];\;E_{\alpha }=\alpha_{n}-\alpha ;\;{\hat{U}}_{\alpha }=\left|\frac{u\cdot {\hat{U}}_{v}+{\hat{U}}_{u}\cdot v}{\left\|u+E_{u}\right\|\cdot \left\|v+E_{v}\right\|\cdot \text{sin}\alpha_{n}}\right| \end{array}$$

In general, in the case of least-squares criteria, no point will belong to the regression line, and the direction will be unique from a dataset. In addition, under the preferred minimum zone tolerance criteria threes points determine the straight line of minimum zone, two of them are colinear in the same direction of the solution (see Figure 3). Consequently, the measured angle by *α_m_* can be expressed, the angle from the coordinates indication of the CMM by *α*, the corrected angle by *α_n_*, and the error and uncertainty are noted by *E_α_* and *Û_α_*, respectively in Equation (11). Note that in the minimum zone criteria each pair of colinear points in the direction that defines the straight lines is determined univocally by u and v. In the case of least-squares criteria the associated uncertainty of the indication could be approached by the root mean square residuals. It can be estimated more appropriated from the measurement model and the law of uncertainty propagation for each reference line, see [29]. Nevertheless, when estimating directly from indication by least-squares criteria, no direct separation of error from the whole variability of the CMM can be incorporated to the measurement.

The measurement of a dihedral angle involves two planes of reference definition, of normal vectors *u*,*v*. They are determined previously from the vectorial product of the 2 vectors defined by the four critical points of the minimum zone solution of each face plane of the block. From those normal directions of each plane the angle by Equation (12) is calculated. In both cases of the angle and the dihedral angle, it is presumed that measuring precisely very small angles close to zero from the former Equations (11) and (12) can be difficult. Uncertainty *Û_α_* increases as the *α_m_* decreases. In these cases the resolution limit to discriminate an angle cannot go beyond the flatness tolerance of the faces that defines it:

(12) $$\begin{array}{l} \begin{array}{cc} & \begin{array}{cc} u_{m}= & u_{m1}\times u_{m2}=u+E_{u}\pm {\hat{U}}_{u}\approx u_{1}\times u_{2}+u_{1}\times E_{2u}+E_{u1}\times u_{2}\pm \left(u_{1}\times {\hat{U}}_{u2}+{\hat{U}}_{u1}\times u_{2}\right)\\ & u_{m1}=(x_{2}-x_{1}+E_{x12}\pm {\hat{U}}_{x12},y_{2}-y_{1}+E_{y12}\pm {\hat{U}}_{y12},z_{2}-z_{1}+E_{z12}\pm {\hat{U}}_{z12})\\ & u_{m2}=(x_{4}-x_{3}+E_{x34}\pm {\hat{U}}_{x34},y_{4}-y_{3}+E_{y34}\pm {\hat{U}}_{y34},z_{4}-z_{3}+E_{z34}\pm {\hat{U}}_{z34})\\ & u_{1}=(x_{2}-x_{1},y_{2}-y_{1},z_{2}-z_{1});\;u_{2}=(x_{4}-x_{3},y_{4}-y_{3},z_{4}-z_{3})\\ & u=u_{1}\times u_{2}\;;\;E_{u}=u_{1}\times E_{u2}+E_{u1}\times u_{2}\;;\;{\hat{U}}_{u}=\left|u_{1}\times {\hat{U}}_{u2}+{\hat{U}}_{u1}\times u_{2}\right| \end{array}\\ & \begin{array}{cc} v_{m}= & v_{m1}\times v_{m2}=v+E_{v}\pm {\hat{U}}_{v}\approx v_{1}\times v_{2}+v_{1}\times E_{v2}+E_{v1}\times v_{2}\pm \left(v_{1}\times {\hat{U}}_{v2}+{\hat{U}}_{v1}\times v_{2}\right)\\ & v_{m1}=(x_{6}-x_{5}+E_{x56}\pm {\hat{U}}_{x56},y_{6}-y_{5}+E_{y56}\pm {\hat{U}}_{y56},z_{6}-z_{5}+E_{z56}\pm {\hat{U}}_{z56})\\ & v_{m2}=(x_{8}-x_{7}+E_{x78}\pm {\hat{U}}_{x78},y_{8}-y_{7}+E_{y78}\pm {\hat{U}}_{y78},z_{8}-z_{7}+E_{z78}\pm {\hat{U}}_{z78})\\ & v_{1}=(x_{6}-x_{5},y_{6}-y_{5},z_{6}-z_{5});\;v_{2}=(x_{8}-x_{7},y_{8}-y_{7},z_{8}-z_{7})\\ & v=v_{1}\times v_{2};\;E_{v}=v_{1}\times E_{v2}+E_{v1}\times v_{2};\;{\hat{U}}_{v}=\left|v_{1}\times {\hat{U}}_{v2}+{\hat{U}}_{v1}\times v_{2}\right| \end{array} \end{array}\\ \begin{array}{l} \text{cos}\alpha_{m}=\frac{u_{m}\cdot v_{m}}{\left\|u_{m}\right\|\cdot \left\|v_{m}\right\|}\approx \frac{u\cdot v}{\left\|u_{nm}\right\|\cdot \left\|v_{nm}\right\|}+\frac{u\cdot E_{v}+E_{u}\cdot v}{\left\|u_{nm}\right\|\cdot \left\|v_{nm}\right\|}\pm \frac{u\cdot {\hat{U}}_{v}+{\hat{U}}_{u}\cdot v}{\left\|u_{m}\right\|\cdot \left\|v_{m}\right\|}=\frac{u\cdot v+u\cdot E_{v}+E_{u}\cdot v}{\left\|u_{nm}\right\|\cdot \left\|v_{nm}\right\|}\pm \frac{u\cdot {\hat{U}}_{v}+{\hat{U}}_{u}\cdot v}{\left\|u_{m}\right\|\cdot \left\|v_{m}\right\|}\\ \text{cos}\alpha_{m}=\text{cos}(\alpha_{nm}\pm U_{\alpha })\approx \text{cos}(\alpha_{nm})\mp \text{sin}(\alpha_{nm})U_{\alpha };\;so\\ \alpha_{n}={\text{cos}}^{-1}\left[\frac{u\cdot v+u\cdot E_{v}+E_{u}\cdot v}{\left\|u+E_{u}\right\|\cdot \left\|v+E_{v}\right\|}\right];\;E_{\alpha }=\alpha_{n}-\alpha ;\;{\hat{U}}_{\alpha }=\left|\frac{u\cdot {\hat{U}}_{v}+{\hat{U}}_{u}\cdot v}{\left\|u+E_{u}\right\|\cdot \left\|v+E_{v}\right\|\cdot \text{sin}\alpha_{n}}\right|\\ \alpha_{m}=\alpha +E_{\alpha }\pm {\hat{U}}_{\alpha }=\alpha_{n}\pm {\hat{U}}_{\alpha } \end{array} \end{array}$$

## 6. CMM Error Model of Roundness or Circularity

The maximum permissible length error or the probe *E_P,MPE_* is estimated through the standard 10360-5 by measuring the roundness defect of a calibrated artefact of known roundness. The roundness criterion has been, ordinarily, the least-squares circle [30] but, according to ISO 1101 [31], the minimum zone tolerance is a preferred criteria of form tolerance. The probe error must be considered in form deviation measurements. In the same manner than a length measurement is aimed to be expressed in terms of the best value and an interval of uncertainty, the roundness expression should include its uncertainty. Roundness of circular shapes will usually be a small fraction of the diameter. Applying the overall error bounds of the ISO CMM verification directly to the roundness error seems to be useless, when not counterproductive. For instance, considering a CMM machine with a *E_L,MPE_* of ±0.006 mm, and a repeatability of 0.003 mm, a 50 mm diameter circle with a indicated roundness of 0.005 mm is measured (output of the machine, or calculated from the best coordinate indication). Giving a confidence interval to the roundness of 0.005 mm, which cannot obviously be based on the *E_L,MPE_*, ±0.006 mm. This can lead to the conclusion that the most probable value is 0.005 mm and the interval of estimated error is so large that the CMM can be hardly trustable about the results in any way. A second approach to transmit the roundness measurement is to give the CMM indication after bias correction, adding the repeatability of the machine as an estimation of the uncertainty (in addition to other possible contributions from temperature, operator, etc.). At least, it would be possible to offer the indicated value with some confidence based on a proper machine setup, CMM repeatability, and the repeatability conditions of the measurement.

**Figure 5:**
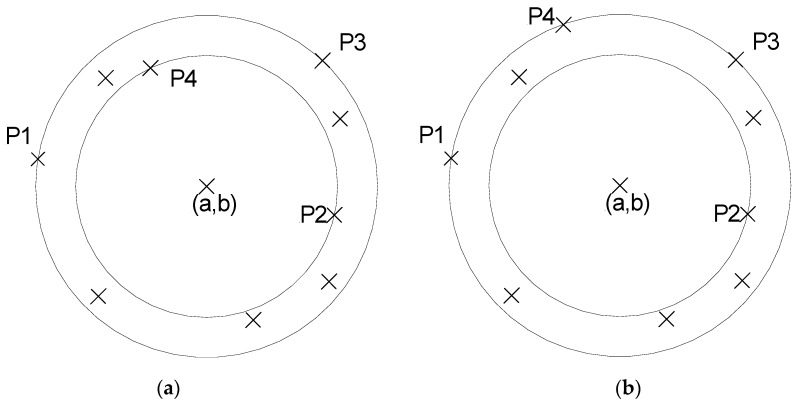
Roundness under minimum zone tolerance criteria, (**a**) configuration (2–2); (**b**) configuration (3–1).

In accordance with ISO 1100, roundness of a feature is the minimum radii difference of two concentric circles that confine the feature between them. The feature is the surface from which the form tolerance is evaluated. In digital machines the feature is sampled in a continuous or discrete probe touching. From this finite set of points, the minimum zone roundness problem is normally determined by four points (see Figure 5) and contains at least one point in the outer and inner solution circles that define the minimum zone [32]. By indicating p_1_(x_1_,y_1_) and p_2_(x_2_,y_2_) those two points, by their respective CMM coordinates indication, the measured minimum zone roundness *MZR_m_* can be expressed, whose solution centre is (*a*,*b*), by Equation (13). The error E fits the mean term of the error (bias or systematic in average), as provided by an accurate roundness algorithm. The non-explained error variability is contained in the uncertainty contribution *Û.* Finally, *MZR* indicates the direct minimum zone calculation from the CMM coordinate indication.

(13) $$MZR_{m}=MZR+E\pm \hat{U}=\sqrt{{\left(x_{1}-a\right)}^{2}+{\left(y_{1}-b\right)}^{2}}-\sqrt{{\left(x_{2}-a\right)}^{2}+{\left(y_{2}-b\right)}^{2}}+E\pm \hat{U}$$

Note that the measurement model is also based on the distance or metric L2 between points of the solution circles and the centre (*a*,*b*). Therefore, those distances with their respective estimations of error in the model can be expressed and its effect on the complete model or the error propagation, by Equation (14). In this case, the error *E_x1a_* is not the realization of a physical length measured and aligned along the X axis. The centre is a calculated point; therefore, the error *E_x1a_* and the corresponding uncertainty *U_x_* estimation are applied with the same vectorial formalism, but without a physical realization that was represented in the former length model of Section 3.

(14) $$\begin{array}{l} MZR+E\pm \hat{U}\\ =\sqrt{{\left[(x_{1}-a)+E_{x_{1}a}\pm {\overset{⌢}{U}}_{x_{1}a}\right]}^{2}+{\left[(y_{1}-b)+E_{y_{1}b}\pm {\overset{⌢}{U}}_{y_{1}b}\right]}^{2}}-\sqrt{{\left[(x_{2}-a)+E_{x_{2}a}\pm {\overset{⌢}{U}}_{x_{2}a}\right]}^{2}+{\left[(y_{2}-b)+E_{y_{2}b}\pm {\overset{⌢}{U}}_{y_{2}b}\right]}^{2}}\\ \;={\left[{\left(x_{1}-a+E_{x_{1}a}\right)}^{2}+{\left(y_{1}-b+E_{y_{1}b}\right)}^{2}\pm 2(x_{1}-a+E_{x_{1}a}){\overset{⌢}{U}}_{x_{1}a}\pm 2(y_{1}-b+E_{y_{1}b}){\overset{⌢}{U}}_{y_{1}b}\pm {{\overset{⌢}{U}}^{2}}_{x_{1}a}\pm {{\overset{⌢}{U}}^{2}}_{y_{1}b}\right]}^{1/2}\\ \;-{\left[{\left(x_{2}-a+E_{x_{2}a}\right)}^{2}+{\left(y_{2}-b+E_{y_{2}b}\right)}^{2}\pm 2(x_{2}-a+E_{x_{2}a}){\overset{⌢}{U}}_{x_{2}a}\pm 2(y_{2}-b+E_{y_{2}b}){\overset{⌢}{U}}_{y_{2}b}\pm {{\overset{⌢}{U}}^{2}}_{x_{2}a}\pm {{\overset{⌢}{U}}^{2}}_{y_{2}b}\right]}^{1/2} \end{array}$$

Considering the Taylor expansion (Equation (15)) and providing small uncertainty *Û* with respect to radii, the expression results as follows (Equation (16)):

(15) $$\varepsilon <<f\to \sqrt{\left(f+\varepsilon \right)}=\sqrt{f}{\left[1+\frac{\varepsilon }{f}\right]}^{1/2}\approx \sqrt{f}\left[1+\frac{\varepsilon }{2f}\right]=\sqrt{f}+\frac{\varepsilon }{2\sqrt{f}}$$

(16) $$\begin{array}{l} {\overset{⌢}{U}}^{2}<<\overset{⌢}{U}<<\sqrt{\left(x-a+E_{xa})+(y-b+E_{yb}\right)};\Rightarrow \\ {\left[{\left(x-a+E_{xa}\right)}^{2}+{\left(y-b+E_{yb}\right)}^{2}\pm 2{\overset{⌢}{U}}_{xa}(x-a+E_{xa})\pm 2{\overset{⌢}{U}}_{ya}(y-b+E_{yb})\right]}^{1/2}=\\ \sqrt{{\left(x-a+E_{xa}\right)}^{2}+{\left(y-b+E_{yb}\right)}^{2}}{\left[1\pm \frac{2{\overset{⌢}{U}}_{xa}(x-a+E_{xa})+2{\overset{⌢}{U}}_{yb}(y-b+E_{yb})}{\left[{\left(x-a+E_{xa}\right)}^{2}+{\left(y-b+E_{yb}\right)}^{2}\right]}\right]}^{1/2}\\ \approx \sqrt{{\left(x-a+E_{xa}\right)}^{2}+{\left(y-b+E_{yb}\right)}^{2}}\left[1\pm \frac{2{\overset{⌢}{U}}_{xa}(x-a+E_{xa})+2{\overset{⌢}{U}}_{ya}(y-b+E_{yb})}{2\left[{\left(x-a+E_{xa}\right)}^{2}+{\left(y-b+E_{yb}\right)}^{2}\right]}\right]\\ =\sqrt{{\left(x-a+E_{xa}\right)}^{2}+{\left(y-b+E_{yb}\right)}^{2}}\pm \frac{{\overset{⌢}{U}}_{xa}(x-a+E_{xa})+{\overset{⌢}{U}}_{yb}(y-b+E_{yb})}{\sqrt{{\left(x-a+E_{xa}\right)}^{2}+{\left(y-b+E_{yb}\right)}^{2}}}\\ =\sqrt{{\left(x-a+E_{xa}\right)}^{2}+{\left(y-b+E_{yb}\right)}^{2}}\pm {\overset{⌢}{U}}_{\text{pc}}\cdot r_{\text{pc}}\\ \text{For a circle of center}\;c=(a,b)\;\text{and a point of the circle}\;p=(x,y)\\ \text{Where}\;{\overset{⌢}{U}}_{\text{pc}}=\left({\overset{⌢}{U}}_{xa},{\overset{⌢}{U}}_{yb}\right);\;r_{\text{pc}}=\frac{\left(x-a+E_{xa},\;y-b+E_{yb}\right)}{\sqrt{{\left(x-a+E_{xa}\right)}^{2}+{\left(y-b+E_{yb}\right)}^{2}}} \end{array}$$

In addition, when the error *E* is much smaller than the radii Equation (17) is obtained:

(17) $$\begin{array}{l} E^{2}<<E<<\sqrt{\left(x-a)+(y-b\right)}\\ \sqrt{{\left(x-a+E_{xa}\right)}^{2}+{\left(y-b+E_{yb}\right)}^{2}}\approx \sqrt{{\left(x-a\right)}^{2}+2E_{xa}{\left(x-a\right)}^{2}+{\left(y-b\right)}^{2}+2E_{yb}{\left(y-b\right)}^{2}}\\ \approx \sqrt{{\left(x-a+E_{xa}\right)}^{2}+{\left(y-b+E_{yb}\right)}^{2}}+\frac{2E_{xa}(x-a)+2E_{yb}(y-b)}{2\sqrt{{\left(x-a\right)}^{2}+{\left(y-b\right)}^{2}}}=\sqrt{{\left(x-a\right)}^{2}+{\left(y-b\right)}^{2}}+E_{\text{pc}}\cdot R_{\text{pc}}\\ \text{For a circle of center}\;c=(a,b)\;\text{and a point of the circle}\;p=(x,y)\\ \text{Where}\;E_{\text{pc}}=\left(E_{xa},E_{yb}\right);\;R_{\text{pc}}=\frac{\left(x-a,\;y-b\right)}{\sqrt{{\left(x-a\right)}^{2}+{\left(y-b\right)}^{2}}} \end{array}$$

Note that the roundness calculation is based on two critical points, namely 1,2, in the inner and outer circles respectively (see Figure 5). Finally, considering Equations (16) and (17) on Equation (14), the *MZR* by Equation (18) can be expressed:

(18) $$\begin{array}{l} \begin{array}{cc} {MZR}_{m12} & =MZR_{12}+E_{12}\pm {\hat{U}}_{12}\\ & =\left[\sqrt{\left(x_{1}-a)+(y_{1}-b\right)}-\sqrt{\left(x_{2}-a)+(y_{2}-b\right)}\right]+\left(E_{1c}\cdot R_{1c}-E_{2c}\cdot R_{2c})\pm ({\overset{⌢}{U}}_{1c}\cdot r_{1c}-{\overset{⌢}{U}}_{2c}\cdot r_{2c}\right) \end{array}\\ \text{In a first order approach}\\ E_{12}=E_{1c}\cdot R_{1c}-E_{2c}\cdot R_{2c}\;\text{by}\;(17)\\ {\overset{⌢}{U}}_{12}=\left|{\overset{⌢}{U}}_{1c}\cdot r_{1c}-{\overset{⌢}{U}}_{2c}\cdot r_{2c}\right|\;\text{by}\;(16) \end{array}$$

The proposed roundness model expresses the measurement result by the nominal calculation from the CMM indication, corrected by the difference of the radii length correction. This correction is the vectorial projection of the length correction ***E*** in the direction of the indicated radii ***R***. Finally, the estimated confidence interval to the error is also the difference of the vectorial projection of the radii length interval of confidence ***Û*** projected in the direction of the corrected radii ***r***.

Just like the flatness model, two criteria can be used for the roundness results: the least-squares and the minimum zone tolerance criteria, but the critical points in the envelope circles will also be, in general, different, four in the minimum zone tolerance criteria, and two others in the case of least-squares, with different expected results. From the fact that the expression of the minimum zone tolerance can be obtained from four points, two in the outer envelope circle and two in the inner one, four different possible estimations are available for error and uncertainty estimation from the original dataset. As a direct consequence of a dataset, the better estimation of roundness by Equation (19) can be proposed. The nominal *MZR* is the same value for all four samples, but not for the error and uncertainty. Under the same previous considerations of the flatness model, the mean error from the different configurations (*c* = 4) is proposed as the best estimation for the error, and derived from its variability the best estimation of uncertainty.

(19) $$\begin{array}{l} MZR_{m}=MZR+\frac{1}{c}\sum_{ii}^{c}E_{i}\pm 2\sqrt{\frac{c-1}{c-3}}\frac{s}{\sqrt{c}}=MZR+E\pm {\hat{U}}_{k=2}\;\mathrm{where}\;s=\sqrt{\frac{{\left(E_{i}-\bar{E}\right)}^{2}}{c-1}} \end{array}$$

## 7. Interpretation of Error and Uncertainty in the Model

The error model by i-axis *E_i_* = *A_i_* + *B_i_L* admits a physical interpretation of its contributors. Already mentioned in Section 2, the ordinate at the origin *A* in the ISO model of CMM verification, ±*E_L,MPE_* = ±(*A* + *B·L*), is the prediction bound or maximum expected error with (*L* → 0); therefore, in the limit it should be the error of the coordinate of a point in an hypothetical bilateral measurement. Consequently, it should be of the same order of magnitude of the repeatability (measured in one-sided or unilateral probing in this case). This will be shown in the experimental Part II. In the ISO 10360-2 approach of machine verification an average null correction to the indication of the CMM coordinates is applied, after systematic error or bias correction by the machine maker’s. That should mean that after correction of any known bias the coordinate determination in any direction of probing has null error. As a result, in a first order approach the indication is accurate. This approach is valid considering the null correction of a grand average of different samples measuring in all possible directions. Unfortunately, a real measurand requires measuring just by a particular direction by the normal to the surface.

In the proposed model by axis *A_i_* is not the prediction bound, but the ordinate at the origin of the linear model, or the mean error with *L* = 0 measuring in the i direction. Therefore, in addition to the mean error by axis, it must be considered the prediction bounds of the error variability that is a contribution to uncertainty, given by *Û_i_*. The interpretation of *A_i_* includes the effects of the parallelism error of the gage blocks and the probe form error. The length realization of the gage block is the distance from any point of a face to the opposite face. The parallelism error of the faces has limits which are specified by ISO 3650, but the regression plane of reference can be biased when a small sample of points is used, when they are not evenly spread around the assigned value of the calibrated standard. Another contribution to *A_i_* is the form error of the probe. The probe correction considers the centre offset, and in a bilateral measuring that error is practically cancelled in a very basic reversal technique. Nevertheless, the imperfection of the probe contact surface in each direction with respect to the perfect average sphere of the tip is just bounded, but not corrected, by the error of form of the probe. As a consequence, it represents a direct bias depending on the direction of measurement.

Note that the careful alignment of the block with the physical axis should be corrected by the normal to the reference surface, so that the measurement is done in accordance with the realization of the length that the standard represents, following similar ISO 10360-2 techniques. In addition, the mean value measuring from one face with respect to the other is averaged in a reverse method that not only compensates the slight error of parallelism [33] of the gage block faces, but also cancels its uncertainty [34].

The slope of the model by i-axis *B*_i_ admits the interpretation of an overall angle error due to the misalignment of the scale and the carriage displacement. The volumetric error (up to 21 type contributors) can be classified in linear terms, angular terms and squareness terms [35]. The angular terms have a main origin in the Abbé error, due to the distance of the probe to the scale in its displacement, which can be approached by a linear model [35]. The squareness errors between axes also propagate linearly with the length of measurement [2]. The slope of the error versus the length measured by the i-axis is *B_i_*. The angular error grows linearly with the length of measurement. A general expression of the angular error is given in [35]. This can be adapted to our formulation by Equation (20), where **θ** denotes the angular pitch error by each axis, and **D** is the matrix of Abbé distances of the measuring head to the axis scale:

(20) $$\begin{array}{cc} B=D\cdot \theta ; & \left[\begin{array}{c} B_{x}\cdot L_{x}\\ B_{y}\cdot L_{y}\\ B_{z}\cdot L_{z} \end{array}\right]=\left[\begin{array}{ccc} 0 & -D_{y} & D_{z}\\ D_{x} & 0 & -D_{z}\\ -D_{x} & D_{y} & 0 \end{array}\right]\left[\begin{array}{c} \theta_{x}\\ \theta_{y}\\ \theta_{z} \end{array}\right] \end{array}$$

After considering the average explained variability of the linear model, the non-explained variability in the regression model can be incorporated into the uncertainty through its variance. This is the approach used when the overall estimation *E_L,MPE_* is taken as a first estimation of the machine uncertainty [6], under repeatability conditions. In the case of the proposed model, the non-explained uncertainty is sensibly lower because the mean error is incorporated into the measurement evaluation model [25]. For a proper estimation of the uncertainty in other valid conditions of the CMM operational range, all other factors that introduce variability should vary in the experimentation [6,36]. Considering the variability of the error a random variable, and due to the real interdependence of CMM hardware and its potential effect in the error of the coordinates, covariance in the error between axes can be expected. Consequently, four out of the seven positions of testing using the ISO 10360-2 verification test are diagonal directions in the cube of the volume of measurement. For the proposed model that only evaluates variances by axis, the issue is whether the variability of error is infra-evaluated or not. In the experimental Part II of this work this issue will be addressed with positive results supporting the proposed model.

Considering the uncertainty by axis estimated by the proposed model, the uncertainty by the law of propagation of uncertainty can also be estimated under the initial hypothesis of independent variables by Equation (21).

It is remarkable that the uncertainty model resulting from the error model of length *U* does not depend on the length itself, but on the direction of measurement (Equation (22)). This means that the uncertainty does not grow with length by axis in a first-order approach. This is well in accordance with the homoscedastic behaviour of error that has been found in the experimental results of the Part II, where the error variability by axis is approximately constant. Note that the independence of *x_1_*, *x_2_*, *y_1_*, *y_2_*, *z_1_*, *z_2_* could be alike when only one axis varies at a time. The independence of *x_1_* and *x_2_*, for instance, is supported by following good practices of bilateral measurement, and it follows the recommendations of ISO 10360-2.

(21) $$\begin{array}{l} u^{2}=\sum_{i=1}^{N}\sum_{j=1}^{N}\frac{\partial f}{\partial x_{i}}\frac{\partial f}{\partial x_{j}}u(x_{i},x_{j})=\sum_{i=1}^{N}{\left(\frac{\partial f}{\partial x_{i}}\right)}^{2}u^{2}(x_{i})+2\sum_{i=1}^{N}\sum_{j=1+1}^{N}\frac{\partial f}{\partial x_{i}}\frac{\partial f}{\partial x_{j}}u(x_{i},x_{j})\\ \text{for independent variables},\;\text{null covariances and expression directly expanded uncertainties}\\ U^{2}=\sum_{i=1}^{N}\sum_{j=1}^{N}\frac{\partial f}{\partial x_{i}}\frac{\partial f}{\partial x_{j}}U(x_{i},x_{j})=\sum_{i=1}^{N}{\left(\frac{\partial f}{\partial x_{i}}\right)}^{2}U^{2}(x_{i})\\ \text{With the vectorial expression of a length},\;\varphi \;\text{elevation angle and}\;\theta \;\text{azimuthal angle}\\ \vec{L}=L\text{cos}\varphi \text{cos}\theta \cdot \vec{i}+L\text{cos}\varphi \text{sin}\theta \cdot \vec{j}+L\text{sin}\varphi \cdot \vec{k};\\ \begin{array}{cc} \text{and}\;L^{2} & ={L_{x}}^{2}+{L_{y}}^{2}+{L_{z}}^{2}={\left(L\text{cos}\varphi \text{cos}\theta \right)}^{2}+{\left(L\text{cos}\varphi \text{sin}\theta \right)}^{2}+{\left(L\text{sin}\varphi \cdot \right)}^{2}\\ & ={\left(x_{1}-x_{2}\right)}^{2}+{\left(y_{1}-y_{2}\right)}^{2}+{\left(z_{1}-z_{2}\right)}^{2}; \end{array} \end{array}$$

(22) $$\begin{array}{l} {\left(\frac{\partial L}{\partial x_{1}}\right)}^{2}={\left(-\frac{\partial L}{\partial x_{2}}\right)}^{2}={\left(\text{cos}\varphi \text{cos}\theta \right)}^{2}={\left(\frac{\left(x_{1}-x_{2}\right)}{\sqrt{{\left(x_{1}-x_{2}\right)}^{2}+{\left(y_{1}-y_{2}\right)}^{2}+{\left(z_{1}-z_{2}\right)}^{2}}}\right)}^{2};\\ {\left(\frac{\partial L}{\partial y_{1}}\right)}^{2}={\left(-\frac{\partial L}{\partial y_{2}}\right)}^{2}={\left(\text{cos}\varphi \text{sin}\theta \right)}^{2}={\left(\frac{\left(y_{1}-y_{2}\right)}{\sqrt{{\left(x_{1}-x_{2}\right)}^{2}+{\left(y_{1}-y_{2}\right)}^{2}+{\left(z_{1}-z_{2}\right)}^{2}}}\right)}^{2};\\ {\left(\frac{\partial L}{\partial z_{1}}\right)}^{2}={\left(-\frac{\partial L}{\partial z_{2}}\right)}^{2}={\text{sin}}^{2}\varphi ={\left(\frac{\left(z_{1}-z_{2}\right)}{\sqrt{{\left(x_{1}-x_{2}\right)}^{2}+{\left(y_{1}-y_{2}\right)}^{2}+{\left(z_{1}-z_{2}\right)}^{2}}}\right)}^{2};\\ U^{2}=2{\left(\text{cos}\varphi \text{cos}\theta \right)}^{2}\cdot U{\left(x\right)}^{2}+2{\left(\text{cos}\varphi \text{sin}\theta \right)}^{2}\cdot U{\left(y\right)}^{2}+2{\text{sin}}^{2}\varphi \cdot U{\left(z\right)}^{2} \end{array}$$

The uncertainty of a point location can be expressed under a GUM approach in a vectorial way by the covariance matrix [37]. Under the hypothesis that the CMM axes show the main independent variations, just as the CMM axis directions are the eigenvalues of the covariance matrix, the uncertainty of a point by Equation (23) can be composed. The uncertainty measured through the three main i-axes, *U*(*i*) is taken into account in Equation (23). Note that the hypothesis that the CMM axes are basically eigenvectors of the covariance matrix is equivalent to inferring that the covariance between axes is neglectable in a first-order approach, already discussed above, so that the aggregation of the variability by axis explains most of the total variability. Once more, this will be verified experimentally in Part II. This does not preclude that, when measuring a feature (plane, circle, etc.), there is a remarkable contribution of uncertainty covariance coming from the geometric measurement model of the feature, used as a substitution geometry (see for instance [29,32]).

(23) $$\begin{array}{cc} U^{2} & =2{\left(\text{cos}\varphi \text{cos}\theta \right)}^{2}\cdot U{\left(x\right)}^{2}+2{\left(\text{cos}\varphi \text{sin}\theta \right)}^{2}\cdot U{\left(y\right)}^{2}+2{\text{sin}}^{2}\varphi \cdot U{\left(z\right)}^{2}\\ & ={\left\|\left(\sqrt{2}U(x),\sqrt{2}U(y),\sqrt{2}U(z)\right).\left(\text{cos}\varphi \text{cos}\theta ,\text{cos}\varphi \text{sin}\theta ,\text{sin}\varphi \right)\right\|}^{2}={\left\|U\cdot n\right\|}^{2} \end{array}$$

The former expression can be compared with the uncertainty derived from the measurement of the distance between two points by our proposed model (Equation (24)):

(24) $$\begin{array}{l} Û=U\cdot n=\frac{{\hat{U}}_{x}(A_{x}+(B_{x}+1)\cdot L_{x})+{\hat{U}}_{y}(A_{y}+(B_{y}+1)\cdot L_{y})+{\hat{U}}_{z}(A_{z}+(B_{z}+1)\cdot L_{z})}{\sqrt{{\left(A_{x}+(B_{x}-1)\cdot L_{x}\right)}^{2}+{\left(A_{y}+(B_{y}-1)\cdot L_{y}\right)}^{2}+{\left(A_{z}+(B_{z}-1)\cdot L_{z}\right)}^{2}}}\\ n=\left(\text{cos}\varphi \text{cos}\theta ,\text{cos}\varphi \text{sin}\theta ,\text{sin}\varphi \right)=\frac{\left(A_{x}+(B_{x}+1)\cdot L_{x},\;A_{y}+(B_{y}+1)\cdot L_{y},\;A_{z}+(B_{z}+1)\cdot L_{z}\right)}{\sqrt{{\left(A_{x}+(B_{x}-1)\cdot L_{x}\right)}^{2}+{\left(A_{y}+(B_{y}-1)\cdot L_{y}\right)}^{2}+{\left(A_{z}+(B_{z}-1)\cdot L_{z}\right)}^{2}}} \end{array}$$

In both cases the uncertainty is the contribution of the uncertaint*y U* projected in the direction of measurement **n**. The expanded standard uncertainty *Û_i_* of the length by *i* direction can be directly expressed from the expanded uncertainty of the point *U*(*i*) in the direction of *i* by Equation (25), also evident by the sensitivity coefficients in Equation (22):

(25) $$\begin{array}{l} \mathrm{If}\;L_{i}=i_{2}-i_{1}\;with\;i_{2}>i_{1};\;{{\hat{U}}_{i}}^{2}={\left(1\right)}^{2}U^{2}(i)+{\left(-1\right)}^{2}U^{2}(i)=2U^{2}(i);\;i=\left\{x,y,z\right\}\\ \mathrm{Thus},\;{\hat{U}}_{x}=\sqrt{2}U(x);\;and\;{\hat{U}}_{y}=\sqrt{2}U(y);\;{\hat{U}}_{z}=\sqrt{2}U(z) \end{array}$$

Therefore, the formulation of uncertainty of the proposed model by axis for the length is the same than that obtained from the evaluation by GUM of the two points that define the length, provided that the uncertainty by the three main directions that are the three main independent contributions (eigenvalues of the covariance matrix) at each point. For this reason it can be assumed that the unexplained variability of error in the regression model of Section 3 is the uncertainty. In Part II, the experimental results of the implementation of the model will give evidence of the usefulness and the degree of compliance of this approach in order to improve the expression of different features measurement by a CMM.

## 8. Conclusions

A linear model of error by axis and its aggregation into a feature measurement model has been developed. It extracts useful information from CMM tests by ordinary techniques of gage block measuring. Probed point coordinates are the original measurand of the CMM that are transformed through signal processing and the measurement model into the feature measurement. The proposed model by axis considers the evaluation of the mean error, and a measurement uncertainty contribution with origin in the non-explained error variability. Both are integrated into the feature measurement model. This model provides the conceptual advantage of incorporating the uncertainty contribution of error variability. Obviously, they should be aggregated with the rest of the uncertainty contributions, such as the calibration gage block uncertainty, and other relevant contributions of the workpiece under measurement but, in particular, it is the coefficient of thermal expansion (CTE) that remarkably affects hardware. As already mentioned, a stream to approach the CMM uncertainty estimation is based on a point uncertainty model and Monte Carlo simulation for uncertainty propagation through the measurement model. This requires hard computation efforts and the subsequent verification of the proposed point uncertainty model based on the real observed CMM behavior. Another way of incorporating uncertainty into CMM feature measurement can be accomplished by ISO 15530, which establishes very strict conditions to evaluate uncertainty based on calibrated artefacts in substitution and non-substitution procedures.

In the proposed model, uncertainty estimation rises directly from the observed measurement variability following the verification process techniques with calibrated artefacts for established reference operating conditions. Uncertainty estimation comes from the verification of a calibrated artefact in order to give an error value for correction to the indicating instrument (CMM). It approaches the ordinary verification process of a simpler instrument, like a micrometer, for instance [23].

The expression of the fundamental measurement model of length, but also flatness, angle, and roundness have been developed. The potential use of this methodology to other CMM measurement models is advisable for future works, in particular for form tolerance. The simple, explicit methodology of the Taylor series development around a value is a well-known approach in modeling and it is also used in the approximate expression of the law of uncertainty propagation by GUM. In this work, its direct application to a CMM linear 3D error measurement model and its integration in the feature measurement is developed.

The modeling methodology with other non-linear functions of error could possibly be further investigated. The model establishes a mean value of error from the regression of the machine response and the residual variability as uncertainty. This local approach is a priori valid as long as the error and its variability are much smaller than the magnitude under measurement. Therefore, a local Taylor series development could be possible. In particular, it can be applied in contactless measurement where sensors translate coordinates into 2D images, which is widely used today in engineering and bioengineering domains.

The proposed model of error compensation uses standard techniques of error verification based on a first-order approach of length errors, valid in the testing zone of the volume of measurement and under similar reference operating conditions of measurement. It is presented as an affordable initial alternative to the detailed mapping of coordinate error. Even when the capability of improving performance might be lower compared to coordinate error compensation from more detailed mapping based on rigid body models, the effort is also lower, because it uses the data obtained from affordable and similar well-established techniques applied in the ordinary periodical verification tests of ISO 10360-2. In this sense, such a capability is viewed as a first step to improve CMM error measuring in an industrial environment. This model can contribute to the inclusion of the compensation of errors and a direct estimation of uncertainty of the CMM’s in the industrial environment. The possibility of post-processing the CMM coordinate raw indications exists, out of the processing operated by the closed CMM’s software. Nevertheless, for operative CMM improvement, the proposed model should be incorporated into the machine software. It can be achieved easily just by fixing the parameters of error by axis after periodical tests. The machine would offer an improved indication after mean error correction and its uncertainty estimation as a part of the measurement result. Experimental evidence of the model’s use and results are developed in Part II.
